# Ovarian cancer: imaging in treatment selection and planning with FIGO update

**DOI:** 10.1186/1470-7330-14-S1-O18

**Published:** 2014-10-09

**Authors:** Evis Sala

**Affiliations:** 1 Department of Radiology, Memorial Sloan-Kettering Cancer Center, 1275 York Avenue, New York, NY 10065, USA

## 

The International Federation of Gynecologists and Obstetricians (FIGO) has recently revised the staging of ovarian cancer [1]. It includes a revision of the stage III patients and allotment to stage IIIA1 is based on spread to the retroperitoneal lymph nodes without intraperitoneal dissemination, because an analysis of these patients indicates that their survival is significantly better than those who have intraperitoneal dissemination [1]. The FIGO staging system for ovarian cancer is surgically based. It does not formally include imaging but the FIGO committee encourages the use of imaging techniques if available to assess the important prognostic factors such as disease resectability and lymph node status.

The standard of care for patients with newly diagnosed advanced ovarian cancer has been comprehensive staging laparotomy and primary optimal surgical cytoreduction followed by adjuvant chemotherapy. However, the use of neoadjuvant chemotherapy followed by interval debulking surgery (IDS) as a suitable alternative is supported by multicenter randomized controlled trials [2]. Imaging is therefore of paramount importance in helping triage patients for appropriate management by accurately evaluating the extent and anatomical location of peritoneal spread, which in turn dictates the feasibility of cytoreductive surgery and predicts the likelihood of optimal primary cytoreduction. Optimal cytoreductive surgery (residual disease <1 cm) is a very strong predictor of survival, and even after the threshold for optimal cytoreduction has been reached, it is important to remove as much of the residual tumor as possible.

CT is the primary imaging modality used to stage ovarian cancer. It is complimentary to surgical staging identifying possible sites of unsuspected disease such as pelvic peritoneum, paraaortic nodes, diaphragm and chest [3,4]. The thorax frequently harbors undiagnosed pleural disease at the time of the initial diagnosis which is likely to affect survival even in cases of optimal debulking [5,6]. Accurate imaging helps guide the surgeon to areas of disease that may be difficult to identify surgically. Relative criteria for non-optimally resectable disease have been developed [7]. They include lymph node enlargement above the renal hilum, presence of abdominal wall invasion, parenchymal liver and subcapsular liver metastases, peritoneal implants of >2 cm along the diaphragm, lesser sac, porta hepatis, intersegmental fissure, gall bladder fossa, gastrosplenic, gastrohepatic ligament and small bowel mesentery. However, it is important to realize that these criteria may vary and will depend on the aggressiveness of the surgical procedure and on the performance status of the patient. Therefore, they should only be used as a basis for a multidisciplinary consensus. It is important to note that upper abdominal disease and pleural metastases can be surgically resected, but this requires careful planning as it involves a team of surgeons (e.g. liver surgeons for hepatic resection). There has been a growing awareness of the potential of diffusion weighted magnetic resonance imaging (DW-MRI) in improving the mapping of the extent of ovarian cancer [8,9]. There is also growing evidence that FDG-PET/CT may play a role in pre-operative staging of patients with advance ovarian cancer [10].
